# Cytochrome P450 Gene Families: Role in Plant Secondary Metabolites Production and Plant Defense

**DOI:** 10.3390/jox13030026

**Published:** 2023-07-25

**Authors:** Panchali Chakraborty, Ashok Biswas, Susmita Dey, Tuli Bhattacharjee, Swapan Chakrabarty

**Affiliations:** 1 Department of Genetics, Development and Cell Biology, Iowa State University, Ames, IA 50011, USA; panchali@iastate.edu; 2 Annual Bast Fiber Breeding Laboratory, Institute of Bast Fiber Crops, Chinese Academy of Agricultural Sciences, Changsha 410205, China; 3 Department of Horticulture, Sylhet Agricultural University, Sylhet 3100, Bangladesh; 4 Department of Plant Pathology and Seed Science, Sylhet Agricultural University, Sylhet 3100, Bangladesh; 5 Department of Chemistry, Jahangirnagar University, Dhaka 1342, Bangladesh; 6 College of Forest Resources and Environmental Sciences, Michigan Technological University, Houghton, MI 49931, USA; 7 College of Computing, Department of Computer Science, Michigan Technological University, Houghton, MI 49931, USA

**Keywords:** cytochrome P450, secondary metabolites, chemical diversity, plant defense, stress tolerance

## Abstract

Cytochrome P450s (CYPs) are the most prominent family of enzymes involved in NADPH- and O_2_-dependent hydroxylation processes throughout all spheres of life. CYPs are crucial for the detoxification of xenobiotics in plants, insects, and other organisms. In addition to performing this function, CYPs serve as flexible catalysts and are essential for producing secondary metabolites, antioxidants, and phytohormones in higher plants. Numerous biotic and abiotic stresses frequently affect the growth and development of plants. They cause a dramatic decrease in crop yield and a deterioration in crop quality. Plants protect themselves against these stresses through different mechanisms, which are accomplished by the active participation of CYPs in several biosynthetic and detoxifying pathways. There are immense potentialities for using CYPs as a candidate for developing agricultural crop species resistant to biotic and abiotic stressors. This review provides an overview of the plant CYP families and their functions to plant secondary metabolite production and defense against different biotic and abiotic stresses.

## 1. Introduction

Cytochrome P450s (CYPs) are one of the most prominent families of enzymes, which are the oxidoreductases class, which uses heme-thiolate as a cofactor. CYPs catalyze NADPH- and/or O_2_-mediated hydroxylation reactions in primary and secondary metabolism in various species. CYPs greatly influence the diversity of metabolites. The metabolites are produced via oxidation, reduction, hydroxylation, epoxidation, dealkylation, C-C cleavage, desaturation, decarboxylation, dimerization, isomerization, and ring extension processes [1]. In 1958, the pigment CYP was discovered in the microsomes of rat liver; today, it is known that CYP is also found in plants, microorganisms, insects, and mammals. They exhibit fantastic natural diversity, as evidenced by their presence in a wide range of organisms. The methods for identification and characterization of CYPs varied between the pre-and post-genomic era. In the pre-genomic era, biochemical methods, such as CYP separation from microsomal fractions and CYP activity inhibition, were utilized to study the function of CYPs. Characterizing CYPs using these methods could be difficult because the CYP superfamily comprises a large gene family and numerous isoforms, making it sometimes difficult to predict the substrate of the enzyme [2]. Later, novel approaches to understanding how CYPs are involved in multiple plant processes, including stress responses, were opened up by the development of next-generation sequencing technologies (NGS) and their advancement and affordability. For instance, there are several CYP genes in the genomes of plants. They represent about 1% of the total protein-encoding genes: 245 in Arabidopsis [3], 316 in grapevine [4], 332 in soybean [5], 313 in poplar [6], and 334 in rice [7].

**Figure 1:**
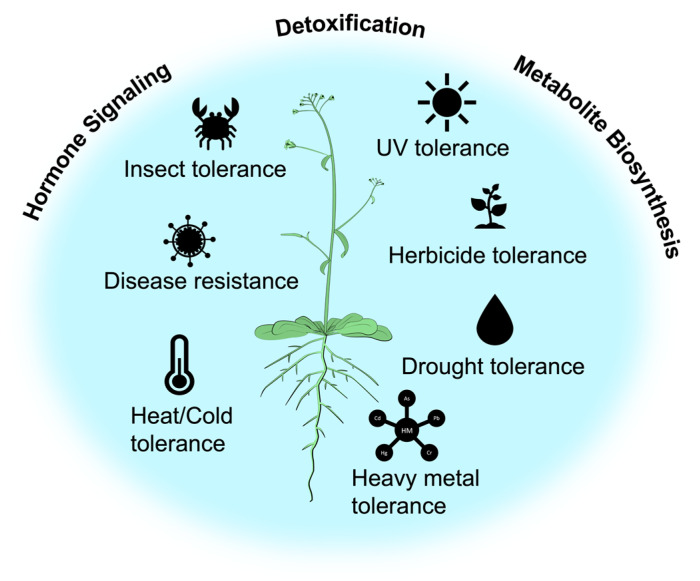
Overview of the role of CYPs in different stress tolerance. CYPs are involved in a wide range of biochemical pathways in plants. Many secondary metabolites, such as alkaloids, flavonoids, and terpenoids, are synthesized by plant CYPs. Plant CYPs are also involved in the biosynthesis of several plant hormones, including auxins, gibberellins, and abscisic acid, and the detoxification of xenobiotics, such as herbicides, pesticides, and pollutants.

There were 318 CYPs counted preliminary in maize, along with initial counts of 372 in sorghum, 142 in papaya, 172 in lotus, 174 in mulberry, 225 in *Bracypodium distachyon*, 270 in tomato, and 399 in potato [8]. Various phylogenetic clans, including single-family and multi-family lineages, have been used to categorize all CYP family proteins of plant origin. These large plant CYPs have developed due to significant evolutionary processes, including gene duplication and diversification. Cytochrome P450 monooxygenases are crucial in producing metabolites. These are necessary for healthy growth and development, and adaptive strategies that define biotic interactions, such as trophic interactions between plants, insects, mammals, fish, and their corresponding pathogens. Plants have accumulated many more CYPs throughout evolution. Throughout their life cycle, plants are subjected to a range of environmental stressors. Plants undergo multiple alterations in their transcriptional, translational, and biochemical levels due to biotic and abiotic stresses, including adjustment to their genetic and protein levels to facilitate adaptation to stressful conditions (Figure 1). It also adjusts enzymatic activities, regulates the concentration of phytohormones to achieve a balanced state, and employs mechanisms to scavenge reactive oxygen species (ROS) to maintain its equilibrium and counteract stress. These adaptations encompass a range of responses to enhance the plant’s resilience and survival in challenging environments. Salicylic acid (SA), jasmonic acid (JA), and ethylene (ET) are three defense signaling chemicals and have complex cross-talk networks that control plant defense [9,10]. Additionally, specific CYP genes connected to the response of plants to stress have been found. These specific CYP genes hold significant promise as potential candidates for developing agricultural species resistant to abiotic and biotic stresses. This review presents an overview of plant CYPs and their functions in plant stress responses.

## 2. Cytochrome P450 Gene Families

Eukaryotes contain a variety of cytochrome P450 monooxygenases comprising a vast and diversified class of enzymes with more than 35,000 members [7]. CYPs participate in various biosynthetic and xenobiotic processes, including incorporating carbon sources, xenobiotic detoxification, and creating secondary metabolites [11,12]. The CYP superfamily is one of the most extensive gene families in plants; it has 245 genes in Arabidopsis, 332 full-length genes, and 378 pseudogenes in soybean [5,13,14]. Initially, the perception of CYP was that of a pigment rather than an enzyme. Later, it was discovered that the specific pigment had striking similarities to heme-protein properties. Because they contain a particular heme group, CYPs can participate in binding carbon monoxide. In plants, the catalytic process involves this specific heme group.

Regarding plants, CYPs were discovered for the first time in the cotton plant [15,16]. After being cloned and phylogenetically categorized, the structures and functions of CYPs from various plants have been revealed. Different CYP types were classified through phylogenetic analysis, utilizing a combination of family numbers and subfamily letters. Paralogs were assigned identical numbers within the same species, while orthologs across different species were assigned shared numbers. For instance, if the amino acid sequence is more than 40% identical to one or more known CYPs, the proteins are grouped into the same CYP family. Moreover, 2 CYPs are regarded as belonging to the same subfamily if they share more than 55% of their genetic makeup. Therefore, unless any particular descriptions are provided, CYPs with more than 97% similarity are assumed to be the same gene [2]. Based on prior findings, the plants’ CYP gene family was divided into eleven clans [7]. Our knowledge of CYP is continually expanding as more genetic data becomes accessible.

CYPs play a pivotal role in a wide range of cellular functions that profoundly impact plant growth and development. CYPs synthesize secondary metabolites, including terpenoids, flavonoids, steroids, alkaloids, phenylpropanoids, glucosinolate, and cyanogenic glycosides. Plant CYP is critical in numerous metabolic processes and can bind to various biological compounds. As a result of these reactions, different fatty acid conjugates, plant hormones, secondary metabolites, lignin, and many protective chemicals are produced [17,18]. Several physiologically active chemicals are attributed to the large variety of CYP genes and their functions [1]. CYP enzymes serve important roles in the evolution and metabolic diversity of plants. Plant CYPs were initially divided into two main clades, the A-type and the non-A-type [19]. Therefore, the A-type group appeared to contain the bulk of P450s producing secondary metabolites. The non-A-type, on the other hand, includes a significantly more divergent set of sequences made up of several distinct clades. They occasionally exhibit a more local similarity to non-plant CYPs than plant CYPs and are involved in the metabolism of lipids or hormones [20].

### 2.1. Non-A-Type CYPs

There are 93 non-A-type, and 7 potential non-A pseudogene has been identified in Arabidopsis. Four branch points were detected in the non-A-type CYP phylogenetic tree based on bootstrap values and intron conservation. However, the phylogenetic tree could not resolve all the deeper branches. The oldest plant CYPs found in Chlamydomonas are the CYP97s and they form a single-family clan [14]. All plants have copies of all three CYP97 subfamilies and are often connected to the synthesis of carotenoids. The CYP74 family also belongs to the non-A-type, which has its own clade. It is found in Rhizobacteria, Cnidaria, and Chordata (in Amphioxus), and is more ancient than plants [21]. However, the evolutionary development of this family is still unclear [14]. Since the CYP74’s main sequence is notably different from other plant CYPs, its position in the evolutionary tree is not firmly determined. The CYP711 is another single-family clade that does not substantially associate with other Arabidopsis clans. The CYP711 clan first appears in green algae, represented by two families, most likely its ancestors. The CYP51 and CYP710 belong to separate clades and are found in fungi, brown algae, and Chlamydomonas. The CYP85 clan is also an important member of non-A-type CYP. This clans consists of the CYP85, CYP87, CYP88, CYP90, CYP702, CYP707, CYP708, CYP716, CYP718, CYP720, CYP722, CYP724 and CYP728 families. Although ancestors of this clan first occur in green algae during the early stages of plant evolution, the families that currently exist in angiosperms first arise in liverworts or bryophytes. The CYP72 clan is made up of the members CYP72, CYP734, CYP714, CYP721, CYP709, CYP735, and CYP715. Liverworts contain the earliest members of this clan, although those families found in angiosperms (CYP72, CYP709, and CYP735) are only found in ferns [14].

### 2.2. A-Type CYPs

In Arabidopsis, there are 152 A-type CYPs and 20 potential A-type pseudogenes. These A-type CYPs are typically distinguished by a single highly conserved intron, M, that is absent from all the other CYP subtypes. Additional conserved introns are ob-served within the various A-type families and subfamilies. However, compared to non-A CYPs, fewer introns are typically present in the A-type. With 52 members in the Arabidopsis genome, the CYP71 family is the most expanded group in the A-type clade. It is the best example of a blooming pattern of gene duplication. The CYP79 and CYP703 are related to the CYP71 because they have the Phe to His substitution in the PERF domain. On the other hand, locating the four families CYP73, CYP77, CYP89, and CYP701 in the CYP71 clan evolutionary trees is challenging. They exhibit distinct intron-exon boundaries compared to other genes within the CYP71 clan. The PERF and heme sequences found in these families exhibit a closer resemblance to the core A-type CYP members, such as the CYP705 and CYP71 families. The conserved M intron is absent from the CYP73, CYP77, CYP89, and CYP701. Although they have different intron arrangements, these four families are more closely linked to the A-type CYPs than to the non-A-type CYPs and non-plant CYPs [14]. With the rapid advancement of DNA sequencing technology, around 200,000 plant CYP gene sequences have been found and compiled in a database [22]. Nevertheless, only a small percentage of plant CYPs have been functionally characterized because screening CYP enzymes for a given catalytic function is expensive. Specifically, for plant CYP enzymes, Wang et al. 2021, developed the PCPCM (plant cytochrome P450 comparative modeling) and PCPLD (plant cytochrome P450 ligand docking) processes, where they used template-based structure prediction and ligand docking, respectively. They conducted cross-docking experiments that effectively demonstrated the validation of PCPLD for virtual screening of CYPs [23].

## 3. Chemical Diversity and Evolution

Plants have substantially greater CYPomes (the cumulative quantity of CYPs present in a specific species) than mammals or microbes. Early in the history of plants, the CYPs began to diversify. In the green algae *Chlamydomonas reinhardtii*, 39 CYP genes were identified, while in *Physcomitrium patens*, a moss species, the number of identified CYP was 71 [24,25]. Green algae, land plants, and a newly identified phylum of sea algae, the Prasinodermophyta, all belong to the plant lineage Viridiplantae. The Prasinodermophyta has been considered to be the oldest diverging phylum within Viridiplantae [26]. Green algae are comprised of charophyte and chlorophyte algae. Twelve recognized algal classes are represented by chlorophytes, whereas charophytes represent five known algal classes. Successful development and evolution of terrestrial plants require changes in biochemistry along with plant architecture [25]. Some CYP families’ emergence during plant evolution is correlated to the appearance of particular metabolite classes, such as carotenoids, phytohormones, oxygenated lipids, or phenylpropanoids. The CYPs are very diverse in green algae. There is no indication of saturation in the count of algal CYP families, suggesting that numerous families are still yet to be discovered. The stark difference between the charophyte and chlorophyte algae can be seen in the fact that only 41 CYP families (around 12%) are shared by these two groups.

**Figure 2:**
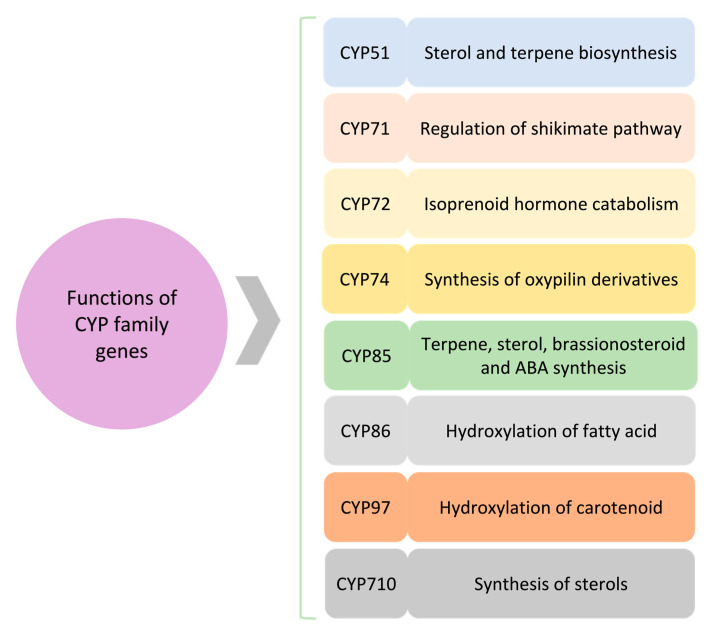
A schematic of the role of major CYP clans. Several CYP clans are involved in plant secondary metabolites and hormone biosynthesis. Synthesis of terpene and sterols is being regulated through CYP51, CYP85, and CYP710. CYP85 also affects hormone regulation, i.e., brassinosteroid and abscisic acid (ABA). CYP86 and CYP97 are involved in fatty acid and carotenoid hydroxylation, respectively. CYP71 is associated with the shikimate pathway, whereas CYP72 and CYP74 impact hormone signaling.

Along with high sequence identity, the presence of fungal CYP55 (nitric oxide synthase) in chlorophytes but not charophytes may indicate lateral transmission of CYP55 after the two algal clades split [27]. Families shared by two or more taxonomic groups and families unique to a particular taxon comprise most of the known CYP families. The estimated numbers of species in each of the nine categories are as follows: gymnosperms: 1000; ferns: 10,500; mosses: 12,000; lycophytes: 1300; hornworts: 200–250; liverworts: 7500; ferns: 10,500; ferns: 12,000; and angiosperms: 300,000. It was believed that CYP clans 51, 74, 97, 710, 711, and 746 comprise of single-family clans. However, this perception has been revised with the annotation of additional algal sequences. It has been discovered that within the 51 clan, there are 3 CYP families unique to green algae, 4 families within the 710 clan, a minimum of 46 families within the 711 clan, and possibly 2 families within the 746 clan. The CYP51, CYP710, CYP97, CYP74, and CYP86 families carry out crucial catalytic processes in metabolism and are conserved in green algae and land plants (Figure 2). Five families (CYP51, CYP710, CYP97, CYP74, and CYP704) are found across several kingdoms. However, green algae do not have CYP704. Despite the recruitment to a specialized pathway and the conservation of duplicates, the families are inclined to be retained as a single copy or at low copy numbers. Gene duplication events have been linked to the growth of the plant CYP superfamily, which was then followed by functional divergence and adaptation to particular metabolic pathways. Whole-genome duplication (WGD) events, which have occurred several times in plant genomes, have helped to enlarge gene families, including the CYP superfamily. One of the largest CYP superfamilies in plants, for instance, the *Arabidopsis thaliana* genome, comprises of more than 250 CYP genes. It has been established that the diversification of plant secondary metabolites and the development of plant defense systems against herbivores and pathogens are connected with expanding CYP superfamily in plants. Utilizing comparative genomics and transcriptomics, a number of research has been conducted on the evolution of plant CYPs [28]. As an illustration, the model tree species *Populus trichocarpa* genome reveals the expansion of CYP families involved in synthesizing lignin and secondary metabolites. Similar growth of CYP families involved in the synthesis of carotenoids and flavonoids was seen in the genome of *Solanum lycopersicum*. These investigations have shed light on the functions and adaptive evolution of plant CYPs. Gene duplication and WGD incidents have aided in the growth of the plant CYP superfamily. Tandem duplication events, which cause the duplication of closely related genes like CYPs, have been demonstrated to be a key mechanism of gene duplication in plants. For instance, simultaneous duplication events within the CYP93 family of *A. thaliana* led to the emergence of two unique subfamilies, CYP93A and CYP93B. While the CYP93B enzymes are engaged in the biosynthesis of flavonoids and lignin, the CYP93A enzymes are involved in synthesizing plant hormones [29].

## 4. Metabolite Biosynthesis

Secondary metabolites are widely present across various plants and play essential roles as compounds for defense and signaling purposes. To date, more than 200,000 secondary metabolites (SMs) have been discovered and characterized. These SMs belong to diverse compound classes, including alkaloids, flavonoids, terpenoids, and glucosinolates [30]. Plants produce different primary or secondary metabolites as a defensive strategy under stress. The CYP gene families have diverse roles in the biosynthesis of metabolic compounds and promote the plant’s development and defense (Table 1). The CYP gene contributes significantly to photosynthesis and photo-protection, which are closely linked to plant growth [31]. Xanthophylls, the yellow pigments, play a vital role in light harvesting during photosynthesis. *CYP97A3* and *CYP97C1* play an active role in the biosynthesis of xanthophylls by catalyzing the hydroxylation of the beta and gamma rings of carotenoids. In the Sorghum plant, specific CYP genes such as *CYP97C1* and *CYP97A3* determine the pathway of lutein. This pathway is crucial for protecting chlorophyll as it facilitates energy absorption and transfer [32]. Moreover, the CYP gene family plays a significant role in auxin biosynthesis in specific plant species. This involvement in auxin biosynthesis is crucial for various tropic behaviors, such as growth towards the light (phototropism) or gravity (gravitropism), as well as root initiation and apical dominance regulation [33]. Furthermore, *CYP734*, *CYP724*, and *CYP90* participate in glycoalkaloids biosynthesis in the Solanaceae family, whereas *CYP74D* genes are essential in the biosynthesis of antimicrobial substances such as oxylipins [34]. These oxylipins are fatty acid derivatives that protect plants against particular stressors.

The biosynthesis of cutin and suberin in plants, like *Symphytum tuberosum* and *A. thaliana,* heavily relies on the active involvement of CYP genes, particularly *CYP86A1*, *CYP86A2*, *CYP86A8*, and *CYP86A33* [35,36,37]. Cutin and suberin are complex biopolymers that protect plants against infections and UV by regulating water evaporation and transpiration. The expression of *CYP72A59v1* and *CYP72A66* is responsible for saponin biosynthesis in response to drought/salt stress in *M. truncatula* [38]. In Arabidopsis, 13-hydroperoxy linolenic acid (13-HPOT) is converted to the plant defense hormone, jasmonic acid, by the allene oxide synthase (AOS) encoded by the *CYP74A1* gene [39]. Meanwhile, *CYP74B4* and *CYP94A52* may be involved in the oxylipin biosynthesis pathway, and *CYP83D3* in the glucosinolates biosynthesis pathway, which may enhance drought/NaCl stress tolerance by minimizing water loss due to stomatal closure. Additionally, it has been observed that the *CYP704G6* gene has a potential role in the biosynthesis of fatty acids and can impact flower development when plants are subjected to drought stress. Meanwhile, the *CYP71A31* and *CYP71AU56* genes are likely responsible for the biosynthesis of indole-3-acetic acid (IAA), a plant hormone associated with various growth processes. These genes may significantly improve plant drought tolerance by enhancing IAA production [38]. The ABA-independent SnRKs (Sucrose non-fermenting 1-related protein kinases) impact the mRNA levels of aquaporins and the enzyme *CYP97B2*. Aquaporins regulate water transport across cell membranes, while *CYP97B2* plays a role in auxin biosynthesis. The activity of ABA-independent SnRKs influences the expression of the gene, thereby affecting water transport and auxin biosynthesis in plants [40]. Under salt stress, *CYP79B2* and *CYP79B3* are induced in Arabidopsis shoots and roots, suggesting a role for the indole-3-acetaldoxime (IAOx) pathway in regulating salt stress responses [40]. Additionally, CYPs facilitate the production of benzoxazinoids, such as 2,4-dihydroxy1,4-benzoxazin-3-one (DIBOA) and 2,4-dihydroxy7-methoxy-1,4-benzoxazin-3-one (DIMBOA), which are essential for enhancing plant defense against diseases, herbivores, and weeds [41,42].

**Table 1:** Potential CYP genes responsible for secondary metabolites biosynthesis and biotic and abiotic stress tolerance.

Plant Species	CYP Genes	Function	Useful Trait	References
*Arabidopsis thaliana*	*CYP97C1*	Lutein biosynthesis	Abiotic stress tolerance	[43]
	*CYP83B1*	Biosynthesis of indole glucosinolates	Abiotic stress tolerance	[44]
	*CYP86A1*	Suberin biosynthesis	Insect resistance	[35]
	*CYP82G1*	Biosynthesis of homoterpene volatiles	Herbivory resistance	[45]
	*CYP86A2*	Biosynthesis of fatty acids	Biotic stress tolerance	[37]
	*CYP83A1* and *CYP83B1*	Glucosinolates biosynthesis	Insect tolerance	[46]
	*CYP86A2, A8*	Cutin biosynthesis	Insect tolerance	[37]
	*CYP51H*, *CYP71A*,*D*, *CYP72A*, *CYP81Q*, *CYP87D*, *CYP88D*,*L*, *CYP93E*, *CYP705A*, *CYP708A*, and *CYP716A,C,E,S,U,Y*	Specialized triterpenes metabolism		[47]
*Solanum tuberosum*	*CYP72A188*, *CYP72A208*	Biosynthesis of steroidal glycoalkaloids	Abiotic stress tolerance	[48]
*Oryza sativa*	*CYP714A3*	Gibberellin biosynthesis	Heavy metal stress resistant	[49]
*Triticum aestivum*	*CYP88A*	Biosynthesis of gibberellin	Heavy metal stress resistance	[49]
	*CYP71*	Biosynthesis of phytotoxin e.g., 2,4-dihydroxy1,4-benzoxazin-3-one (DIBOA) and 2,4-dihydroxy7-methoxy-1,4-benzoxazin-3-one (DIMBOA)	Biotic stress tolerance	[50]
*Pisum sativum*	*CYP88A*	Biosynthesis of gibberellin	Heavy metal stress tolerance	[44]
	*CYP96A15*	Biosynthesis of epicuticular wax	Biotic stress tolerance	[51]
*Panax ginseng*	*CYP71*	Biosynthesis of secondary metabolites, alkaloids, and flavonoids	Heavy metal stress resistance	[52]
*Zea mays*	*CYP71*	Biosynthesis of phytotoxin, e.g., DIBOA and DIMBOA	Biotic stress tolerance	[50]
	*CYP96A15*	Biosynthesis of epicuticular wax	Biotic stress tolerance	[51]
	*CYP1Capsicum annuum*	Synthesis of dehydrin	Biotic stress tolerance	[53]
*Nicotiana benthamiana*	*CYP51H10*	Biosynthesis of triterpenes	Biotic stress tolerance	[54]
*Nicotiana rustica*	*CYP51*	Biosynthesis of triterpene		[54]
*Helianthus tuberosum*	*CYP96A15*	Biosynthesis of epicuticular wax	Biotic stress tolerance	[51]
*Sorghum bicolor*	*CYP79A1* and *CYP71E1*	Biosynthesis of the cyanogenic glucoside	Insect tolerance	[55]
	*CYP79*	Cyanogenic glucosides biosynthesis		[56]
*Catharanthus roseus*	*CYP72A1*	Alkaloid biosynthesis	Disease resistance	[57]
*Ocimum basilicum*	*CYP82D*	Biosynthesis of different secondary metabolites		[58]
*Vitis vinifera*	*CYP75*	Flavonoid biosynthesis		[59]
*Gossypium arboreum*	*CYP706B1*	Alteration of the isoprenoid pathway		[60]
*Symphytum tuberosum*	*CYP86A33*	Biosynthesis of suberin		[36]
*Pogostemon cablin*	*CYP71*, *CYP77*, *CYP81*, *CYP82*	Biosynthesis of sesquiterpenes		[61]
*Citrus* sp.	UV-B-induced CYP gene	Biosynthesis of 3′-hydroxylated flavonoids		[62]
*Cajanus cajan*	*CYP75B165*	Biosynthesis of flavonoids		[63]
*Glycine max*	*CYP82D26*	Isoflavonoid pathway biosynthesis		[64]
Legumes	*CYP93C*	Synthesis of legume specific isoflavonoid		[65]
Land plants	*CYP93B*, *CYP93E*, *CYP93G*	Saponin biosynthesis		[66]
Higher plants	*CYP74A*	Synthesis of allene oxide	Biotic stress resistance	[67]

## 5. Xenobiotic Metabolism and Hormone Regulations

CYPs are one of the main enzymes in charge of detoxifying exogenous molecules in plants and other organisms. Toxins, including pesticides, heavy metals, allelochemicals, organic pollutants, and other pollutants, are frequently exposed to plants. Plants build specialized and coordinated defense mechanisms to survive in unfavorable growing conditions. CYP’s involvement in the biosynthesis of plants’ hormones, lipids, and secondary compounds is better understood than its function in detoxifying exogenous chemicals [68]. On the other hand, they have the potential to serve as a crucial metabolic reservoir for environmental contaminants [69], presenting a promising approach to regulate and control herbicide selectivity and tolerance [70], and could represent a good potential for bioremediation.

The ability of some monocotyledonous plants’ herbicide detoxification system to be induced by a class of synthetic chemicals known as herbicide safeners. Safeners can shield grass fields from herbicide damage without lowering herbicide activity in target weed species. They protect the target plant from herbicide injury while inducing the expression of genes involved in plant defense and detoxification, such as glutathione S-transferases (GSTs) and CYPs. This indicates that safeners use a previously unrecognized signaling pathway to detoxify xenobiotics or endogenous toxins [71]. CYPs mediate the primary metabolism of several important chemical classes of herbicides, frequently resulting in crop selectivity [72]. The connections between the physiological and detoxification roles of various CYP isoforms and herbicide selectivity have been well studied in rice and maize [73,74,75]. The safener responsiveness was primarily attributed to clan 71 and clan 72 members within the CYP family, with fewer outliers in clan 86. Most of the upregulated CYP71 clans in Arabidopsis belong to the family CYP81. In maize, *CYP81A9* and *CYP81A6* have been linked to herbicide sensitivity in sweetcorn cultivars with five different mechanisms of action [76]. Further evidence demonstrated that *Echinochloa phyllopogon* and *Lolium rigidum* are resistant to graminicides and had an increased expression of clan 71 and 72 CYPs [77,78].

Herbicide metabolism is linked to several members of the CYP family. The involvement of CYPs is crucial in the metabolic transformation of target sites, leading to structural changes. Herbicide metabolism is separated into three phases, i.e., conversion (phase 1), conjugation (phase 2), and compartmentation (phase 3). The Phase 1 metabolism of herbicides is mostly mediated by CYPs [79]. In general, there are different criteria which are considered to demonstrate that CYP was involved in the development of resistance. For instance, the position in the microsomal palate, NADPH and oxygen requirements, CO-induced light-reversible inhibition, antibody-mediated inhibition of CYP reductase and CYP inhibitor-induced suppression. It is established that a positive correlation exists between elevated CYP enzyme activity and herbicide resistance in weed plants [79]. Recent research has shown that overexpression of the ginseng-derived *CYP736A12* in Arabidopsis results in resistance to chlortoluron and isoproturon. The functional study of *PgCYP76B93* provides information on using this gene as a marker for genetically modifying multipurpose crop species that can detoxify agrochemicals and environmental toxins [80]. The broader exploration of gene activity and regulation in various xenobiotic metabolizing enzymes has provided a comprehensive understanding of the functioning of plant detoxification systems across multiple species. This information will be highly advantageous in unrevealing the genetics and molecular underpinnings of plant herbicide selection.

Plant CYP enzymes tightly regulate the biosynthesis and catabolism of plant hormones like auxins, gibberellins, cytokinins, and abscisic acid (Table 2). The hydroxylation, epoxidation, and oxidative cleavage of hormones by these enzymes can alter their bioactivity or functions. Recent developments in molecular genetic analysis led to identifying and characterizing genes involved in producing plant hormones. In Arabidopsis, *CYP79B2/B3* and *CYP83* control the production of auxin. Gibberellin metabolism involves several CYPs in different plants, including *CYP88A* and *CYP714A* in Arabidopsis and *CYP701A8* and *CYP714B* in rice. Moreover, the Arabidopsis *CYP735A1* and *CYP735A2* genes code for cytokinin hydroxylases that catalyze trans-zeatins, and the *CYP707A* gene code for an enzyme called ABA 8′-hydroxylase that regulates the amount of abscisic acid in Arabidopsis and barley (Table 2). The polyhydroxysteroid class, known as brassinosteroids (BRs), has been recognized as the sixth class of plant hormones. Numerous CYPs control BR biosynthesis, for instance, *AtCYP72B1* and *AtCYP72C1,* control photomorphogenesis and plant steroid signal transduction by deactivating BR, and *AtCYP85A* controls the production of BR C-6 oxidase. *AtCYP90A1* and *AtCYP90B1* enhance vegetative growth and seed yield by regulating the biosynthesis of BRs. On the other hand, *CYP90B3* and *CYP724B2* catalyze the initial C-22 hydroxylation reactions in the BR biosynthesis pathway of Tomatoes [81].

## 6. Plant Defense against Herbivory and Pathogenicity

Biotic stress from living organisms—such as insects, pathogens, weeds, etc.—results in plant damage by disrupting the general dynamics of plants. These stresses deprive the host plant of the nutrients and minerals it needs, which can, in some severe circumstances, result in plant death. In addition to interfering with plant growth, biotic stressors also impact seed quality, root rot, and crop yield. Terpenoids, phytoalexins, alkaloids, cyanogenic glucosides, etc., are vital for a plant’s ability to respond to biotic stresses—they are synthesized proportionally by CYPs.

### 6.1. Cytochrome P450 in Plant-Pathogen Interaction

Among the various biotic stresses, the disease is regarded as the most predominant. Fungi, viruses, and bacteria play a substantial role in causing disease in plants.

#### 6.1.1. Cytochrome P450 in Plant-Bacteria Interaction

In Arabidopsis, the *AtCYP76C2* gene causes faster cell death to cope with stress caused by *Pseudomonas syringae* because it prevents the spread and proliferation of the pathogens [91]. The hypersensitive response (HR), driven by a *P. syringae* infection in *A. thaliana,* increases the expression of the *CYP76C2* gene. The *CYP76C2* gene expression is linked to injury, aging cell culture, and leaf senescence [92]. Phytoalexins are low molecular weight antibacterial chemicals with a wide range of structural variations produced rapidly by many plants in response to bacterial and fungal attacks. In Arabidopsis, *CYP71* and *CYP79* are reported to be associated with producing the phytoalexin ‘camalexin’ [93]. In *Capsicum annuum,* *CaCYP1* is crucial for recognizing HR for safeguarding the plant against infection by *Xanthomonas axonopodis*. The *CYP93C* gene facilitates plant tolerance to disease stress by increasing the biosynthesis of isoflavonoids for plants, including *Medicago truncatula*, *Glycyrrhiza echinata*, *Glycine max*, and *Cicer arietnum*. Other *CYP* genes, such as *CYP81E1*, *CYP81E3*, *CYP81E7*, and *CYP93A1,* are also reported as significant players in the production of isoflavonoids [94]. In potatoes, the pathogen-inducible divinyl ether synthase from the CYP74 family produces the antibacterial substance oxylipin [34]. Oxylipins are derived from polyunsaturated fatty acids, play a crucial role in plant physiology. Among the major oxylipins produced by the plant, methyl jasmonate and jasmonic acid are particularly prominent. These compounds are primarily synthesized in response to physical injury and serve as key components in the plant’s defense against diseases. *Withania somnifera* has been studied to understand the role of CYPs in plant defense. The stalk and root have been found to express large amounts of the elicitor-responsive genes, including *WsCYP98A* and *WsCYP76A*. The role of *WsCYPs* in metabolite biosynthesis and defense against bacteria has also been reported in a prior study [95].

#### 6.1.2. Cytochrome P450 in Plant-Fungi Interaction

The evolution of fungal pathogenicity has been linked to the diversification and extension of the CYP family. When pathogens attack host plants, CYP enzymes respond by inhibiting the formation of antibiotic phytoalexins [96]. Pisatin is a kind of phytoalexin produced by Pea, has been inactivated by CYPs in response to fungal attack [97]. Numerous crop plants are affected by the vascular wilt disease caused by the fungus *Fusarium oxysporum*. Antagonistic *F. oxysporum* strains defend plants from fungal attacks. In Lettuce, the pathogenic *formae specialis* of *F. oxysporum* expresses *CYP505* in the host plant, which hydroxylates saturated fatty acid and helps plant tolerance. Lauric, palmitic, and stearic acids in the cortical cell membranes of the plant can be mono-hydroxylated by *CYP505A1*, and these hydroxylated substances may trigger the plant’s defensive mechanism [98]. The *pepCYP* gene participates in the defense mechanism against the pathogenic fungus *Colletotrichum gloeosporioides* which attack in the pepper. A CYP found in pepper called *CYP89,* is essential for the plant’s defense against pathogen infestations [53]. CYPs are involved in the signaling pathways that are triggered by jasmonic acid and methyl jasmonate. For example, methyl jasmonate activates the *CYP82A3* gene in soybeans, which linked to fungal infections. *N. benthamiana* transgenic line overexpressing the *CYP82A3* gene is highly resistant to gray mold and black shank disease [99]. Growing evidence shows that CYP enzymes are essential for plant defense against pathogenic fungi. Biochemical roles of CYPs associated with the metabolism of plant triterpenes have also been identified. *CYP51H10* was required to synthesize anti-microbial oleanane-triterpene saponins (avenacins), which gave oats disease resistance against the fungi that infected the roots [100].

### 6.2. Cytochrome P450 in Plant-Insect Interaction

The number of CYP genes in plant genomes has expanded more significantly than in insect and vertebrate genomes. Numerous plant pathways evolved to produce ecologically significant secondary metabolites as defenses against insects. Maize has been developed into an important model species in the past two decades for research on physiological and molecular aspects of plant–insect interactions. The comprehensive defense mechanisms in maize have been studied in response to caterpillar feeding. The results revealed that the maize plant accumulates secondary metabolites for defense [101]. The simulated herbivory increases the expression of *CYP79A61,* and produces aldoxime, and phenyl acetaldoxime, which helps maize to defend against insect infestation [102]. In the arms race against herbivores, plants constantly evolve new defense chemicals to survive. One such class of specialized chemicals is saponins. The structure of saponins comprises a hydrophobic triterpenoid backbone with one or more hydrophilic saccharide groups. Saponins are poisonous or repellent for some insects, mollusks, fungi, and other microbes. In *Barbarea vulgaris*, a tandem repeat of eight *CYP72A* CYP colocalizes with a quantitative trait locus (QTL) for saponin accumulation. The function of *CYP72A552* has evolved to catalyze the production of saponins based on hederagenins, which mediate plant defense against herbivores [103].

A multimeric complex containing a 13-lipoxygenase, allene oxide synthase (AOS), and allene oxide cyclase was found in *A. thaliana*. AOS is a member of the CYP74A family of CYP enzymes. AOS faces competition from hydroperoxide lyase (HPL, *CYP74B*), another CYP found in chloroplasts, for the same substrate. The ability of plants to respond to various biotic stresses depends on their AOS and HPL activities. The accumulation of JA is observed in plants as a reaction to various abiotic and biotic stresses. Additionally, JA is actively involved in wound responses and is a critical component in plant defense mechanisms. The AOS and HPL complexes regulate the production of JA during plant growth and stress response [104].

Insect pests cause physical damage to plants and act as vectors for viruses and bacteria. *CYP* genes have been involved in wound-induced defense. In sorghum, *CYP71E1* and *CYP79A1* enzymes catalyze the conversion of tyrosine, improving pest resistance and food safety [12]. Few plants, including Arabidopsis, have *CYP86A1*, which is involved in the manufacture of suberin that play a vital role against insect stress [35]. In Arabidopsis, the CYP family *PAD3* gene is associated with the production of camalexin, conferring resistance against the green peach Aphid [105]. In *P. trichocarpa*, the synthesis of aldomixes is triggered by the CYP79D gene family under herbivore infection [102]. An unidentified CYP hydroxylase was found to convert cembratriene-ol (CBT-ol) into cembratriene-diol (CBT-diol) in the trichome glands of *N. tabacum*. By suppressing this, CYP increased the concentration of CBT-ol and displayed Aphid resistance [106]. In *Coptis japonica*, an alkaloid called berberine is produced by the *CYP719*, which is harmful to insects, including the Diamondback Moth.

## 7. Plant Abiotic Stress Tolerance

Plants can function normally in optimal conditions but often experience different biotic and abiotic stresses [107]. Exposure to different stresses interferes with their natural growth, leading to a significant loss in agricultural production [108]. It has been discovered that CYPs are involved in hormone signaling, hence governing plant response to different stresses [109,110]. CYPs defend plants from various stresses caused by environmental variables such as drought, heat, salt, and heavy metals [111,112,113]. Furthermore, CYPs play a direct role in plant secondary metabolism, actively by detoxifying external pollutants or those generated as metabolic byproducts in response to stress conditions [107].

### 7.1. Temperature Stress

Temperature stress, either heat or cold stress in plants, results in several undesirable occurrences, such as disruptions in respiration and photosynthesis, decreased cell membrane stability, and protein denaturation, ultimately leading to oxidative damage mediated by ROS [114]. In plants, heat stress leads to the denaturation of proteins by promoting the degradation of cellular proteins. It also affects the membrane’s permeability as plants’ cell walls disintegrate under high heat stress conditions [115]. Many findings indicated that heat or cold stress impacted the expression level of most flavonoid-related transcripts. Expression of *CYP73A*, *CYP75A*, and *CYP75B* genes involved in the production of flavonoid antioxidants such as trans-cinnamate 4-monooxygenase, flavonoid 3′,5′-hydroxylase, and flavonoid 3′-monooxygenase, respectively, was considerably increased in *Lolium perenne* and *Festuca arundinacea* in extreme low and extremely high temperatures [116]. The upregulation of *CYP99A1* and *CYP709C1* in cold-tolerant Sorghum indicates their involvement in low-temperature stress tolerance [117].

It has been reported that during prolonged cold stress in Arabidopsis, the *CYP83A1* gene was found to be more expressed, playing a vital role in flavonoid (phenylpropanoids) metabolism [118]. It was reported in other studies that the *CYP707A* genes in Arabidopsis play a role in increasing the levels of ABA, specifically through the action of ABA 8′-hydroxylases. This increase in ABA levels facilitated the response of Arabidopsis to temperature stress [114,119]. In *Panicum virgatum,* 11 CYPs were differentially expressed under prolonged heat (38/30 °C, day/night, for 50 days), where, for the production of indole alkaloid secologanin, 2 *CYP71A1* genes need to be present [120]. In contrast, the expression of *CYP71* was significantly reduced in the leaves of *Rhazya stricta* when exposed to elevated temperatures (40–42.4 °C) [121]. Under high-temperature stress, *CYP71A23* is responsible for pollen sterility in *Brassica napus* [122] and the expression of this gene increased in *Panicum maximum* [123].

### 7.2. Salinity Stress

Salinity stress refers to the inability of plants to uptake water due to the presence of excessive salts, particularly soluble salts, such as sodium chloride (NaCl), in the soil. The negative impacts of NaCl on plants are primarily due to decreased water availability due to Na^+^ accumulation in soil, increased oxidative stress, and the toxic effects of Na^+^ and Cl^−^ ions [124]. Salt stress affects germination, growth and development, photosynthesis, and yield, eventually leading to death [125]. Plants have developed numerous methods to survive in highly salinized soil. It has also been demonstrated that CYP proteins are crucial in plant responses to salt stress by regulating hormone signaling and maintaining ROS homeostasis [126,127]. For example, expression of the *CYP81D5* in maize and wheat at vegetative and reproductive stages enhanced salinity tolerance through ROS scavenging activity. The salinity stress response is closely associated with the induction of ABA. ABA may reduce transpiration, wilting, water uptake, and stomatal conductance. In contrast, ABA is favorable to maintaining low Na^+^/K^+^ and enhancing the activity of protecting enzymes, which ultimately keeps an intact cell membrane structure and reduces the damage caused by salt [128,129]. It has been reported that *CYP707A* protects plants from salt stress and increases salt tolerance by regulating ABA levels. The over-expression of the *CYP709B3* gene improved salt-stress tolerance in transgenic Arabidopsis [130].

In a separate study, *CYP736B*, the homolog of *CYP736A12*, conferred enhanced salt tolerance by reducing H_2_O_2_ accumulation, increased carotenoid levels, and ABA biosynthesis in Arabidopsis [131]. Similarly, upregulated expression of *CYP709* was observed in salt stressed *Robinia pseudoacacia* [132]. In a salt-tolerant strain of *Physcomitrella patens*, 50 CYPs modified their protein level under salinity stress to lessen cell wall deterioration and scavenge ROS [133]. The expression of *CYP75A3* and *CYP73A11* genes in cotton indicated salt tolerance with a reduced level of hydrogen peroxide and a significant increase in glutathione, ascorbate peroxidase, and proline. It also reported that knocking down these two genes downregulates several stress-responsive genes, making them more susceptible to salt stress [134]. Furthermore, it was revealed that salt stress significantly induced *PtCYP714A3* from *P. trichocarpa*, and transgenic rice demonstrated enhanced salt tolerance [135]. In the mangrove tree *Avicennia officinalis*, *AoCYP94B3* and *AoCYP86B1* were found to reduce the suberin of the roots, thus leading to salt tolerance [136]. Another study revealed that heterologous expression of *CYP94B1* in rice, *Avicennia officinalis*, and Arabidopsis enhances seedling salt tolerance by improving root suberin deposition [137].

### 7.3. Drought Stress

Water or drought stress refers to the insufficiency of moisture, recognized as a critical prerequisite for the optimal growth of a plant. The survival of plants during drought stress relies on the maintenance of cell homeostasis in a water-deficit environment. In response to such stress, plants activate specific hormones as a defense mechanism. ABA is an important plant hormone linked to drought stress and fluctuates dramatically during dehydration and rehydration. Nevertheless, in such scenarios, the catabolism of ABA plays a crucial role in maintaining the balance of ABA levels [138]. During drought stress, the presence of enzyme ABA 8′-hydroxylase (*ABA8Ox*) of the CYP707 family catalyzes the hydroxylation of the ABA, resulting in the formation of phaseic acid (PA) [139]. When maize was exposed to water deficit conditions, the *CYP707A* (*ABA8Ox*) gene was upregulated [140]. Similarly, *CYP707A1* and *CYP707A2* were significantly upregulated in *Arachis hypogaea* and *Populus simonii* under osmotic stress [141,142]. Moreover, it has been demonstrated that Arabidopsis exhibits upregulation of the same genes in response to drought stress [138].

During drought, the *CYP96A8* gene is involved in the lignin biosynthesis of maize leaf, forming the necessary structural materials supporting plant tissues and other functions related to drought tolerance [143]. The plant cuticle is an important structure required for controlling non-stomatal water loss. In Arabidopsis, *CYP86A8* is associated with the omega-hydroxylation of fatty acids and produces the monomer of cutin [144]. In contrast, *CYP86A2* plays a significant role in epicuticular lipids synthesis, including cutin, reducing the cuticle membrane thickness. This reduction enhances water permeability, thereby aiding in drought tolerance mechanisms [37]. The specific role of *CYP86A2* in drought tolerance mechanisms may vary among plant species. However, further research is needed to fully understand the specific mechanisms and the extent of *CYP86A2*’s impact on drought tolerance. During flower development in *M. truncatula*, *CYP704G6* was highly expressed, suggesting it plays a similar role to *CYP704B2* in fatty acid biosynthesis under drought conditions. In water-deficit states, CYPs participate directly or indirectly in the biosynthesis of antioxidants that can reduce oxidative damage [38]. In *Citrus sinensis, CsCYT75B1* is upregulated in drought conditions, and overexpression of this gene is responsible for increased total flavonoid content and antioxidant activity [145]. In Arabidopsis, isoflavone and flavonoid 30-monooxygenase biosynthesis catalyzed by *CYP81E9* and *CYP78A126* may improve plant resistance to drought by lowering scavenge ROS [146]. Some other CYPs are involved in flavonoid biosynthesis, for instance, the CYP75 family regulates flavonoids biosynthesis in grapevines and ferns [6], heterologous expression of *CYP82G24* in *Carthamus tinctorius* induces flavonoid biosynthesis genes [147].

It has been revealed that loss of *CYP85A2* lowered the brassinolide (BL) accumulation and enhanced drought tolerance in Arabidopsis [148]. In another research, overexpression of *SoCYP85A1* indicated drought stress tolerance due to higher castasterone (CS) (the precursor of the BL biosynthesis) accumulations. An increased abundance of CS results in a sharp decrease of BL, which enhances root growth, regulates ROS and controls the expression of genes that respond to stress [149].

### 7.4. Heavy Metal Toxicity

Crop production and quality are significantly hampered by heavy metal contamination of the soil and water, which causes toxicity and stress. Among all the heavy metals, lead, mercury, chromium, arsenic, and cadmium are the most toxic [150]. CYPs play an important role in detoxification when exposed to heavy metal stress [151]. The genes *CYP71* in *P. ginseng*, *CYP2E1* in *Medicago sativa*, *CYP81D8* in *A. thaliana*, *CYP88A* in *T. aestivum,* and *P. sativa* have been associated with heavy metal stress tolerance. In Hg-contaminated soils, *M. sativa* plants that have been genetically altered with the human *CYP2E1* and *GST* genes showed the potential for phytoremediation. Additionally, transgenic *M. sativa* expressing both of these genes showed a synergistic impact that improved metabolism in plants, enabling them to survive mercury exposure [152]. In *P. ginseng*, heavy metal interactions with the cell membrane produced several defense-related genes through the jasmonic acid pathway. The plant also has cross-talk networks with other defense-related pathways [153]. *P. ginseng* exhibited upregulation of *CYP71* when exposed to heavy metals, particularly nickel and cadmium [52]. In maize, the *CYP88A* facilitates the synthesis of gibberellins, which integrates multiple hormone-signaling pathways in response to heavy metal toxicity stress [49].

### 7.5. Herbicide Stress

Herbicide resistance refers to a plant’s innate capacity to withstand while applying herbicides for weed control. The capacity of a species to live and reproduce following exposure to an herbicide at a regular application rate is known as herbicide tolerance. Herbicide exposure may cause a variety of physiological and biochemical reactions in plants. CYPs are involved in the physiological mechanism of hormones and secondary metabolites biosynthesis that confers herbicide resistance [79,154,155]. For instance, the overexpression of *CYP736A12* in Arabidopsis has been found to slightly reduce plant height and contribute to the metabolism of phenylurea herbicides such as chlorotoluron and isoproturon, thus conferring herbicide resistance [156]. Both chlorotoluron and isoproturon, and phenylurea herbicides, actively metabolize the functionally defined CYPs *CYP73A1* and *CYP81B1* from Jerusalem artichoke. Following its identification in Jerusalem artichoke, *CYP76B1* was subsequently discovered to possess the ability to metabolize herbicides belonging to the phenylurea family. CYP71A10, an enzyme derived from soybeans, may metabolize the phenylurea herbicide, chlortoluron, when expressed in yeast. However, it does so with ten times less effectiveness than CYP76B1 [122,157,158,159]. In addition, transgenic Arabidopsis and *Nicotiana tabacum* having rice *CYP81A6* functionally characterized that *CYP81A6* is involved in the betazon metabolism [74]. The *CYP76C1*, *CYP76C2*, and *CYP76C4* genes also demonstrated herbicide tolerance in Arabidopsis. The heterologous expression of Wheat *CYP71C6v1* was responsible for the metabolism of herbicides, including chlorsulfuron, triasulfuron, metsulfuron-methyl, bensulfuron-methyl, and tribenuron-methyl [160]. The gene *CYP81A9* in maize has been identified as responsible for the metabolism of the acetolactate synthase (ALS) inhibitor herbicide nicosulfuron [161]. Maize has also demonstrated CYP-mediated metabolism of herbicides such as tembotrione, mesotrione, dicamba (synthetic auxin), diflufenzopyr (auxin transport inhibitor), and carfentrazone (protoporphyrinogen oxidase inhibitor) through the 4-hydroxyphenylpyruvate dioxygenase (HPPD) inhibitors [75,162,163,164].

## 8. Future Research

CYPs are multifunctional enzymes that contribute significantly to plant stress response, growth, and development processes. Through biosynthesis and regulation, CYPs enable plants to produce and control the levels of hormones, fatty acids, sterols, cell wall components, biopolymers, and other defense chemicals. Many CYPs have been identified, but their specific functions and contribution to the physiological process are still unknown. Understanding the roles of individual CYP is necessary to elucidate their particular biological processes. Future studies can use cutting-edge genomics, transcriptomics, proteomics, metabolomics, and bioinformatics technologies to study CYP family genes. Through genomics and transcriptomics, researchers can identify stress-responsive genes involved in stress response mechanisms and analyze the expression patterns of target CYP genes under diverse environmental conditions and developmental stages. Omics data can provide resources of molecular markers associated with desirable traits. These markers can be used for marker-assisted selection, allowing breeders to identify and select plants with specific traits more efficiently. GWAS has been extensively used to study CYPs in many plant species. GWAS at the epigenome, transcriptome, protein, and metabolic levels for crop breeding might help distinguish the beneficial and detrimental alleles. Metabolomics can provide insights into the metabolic pathways and biochemical processes associated with stress tolerance and productivity. Research on the protein–protein interactions of CYPs with additional enzymes, regulatory elements, and protein complexes would reveal important information about their molecular mechanisms and possible interacting partners in metabolic pathways. To find interacting partners and investigate the dynamics of CYP-mediated protein complexes, methods including yeast two-hybrid experiments, co-immunoprecipitation, and protein–protein interaction networks can be used. Alphafold is a powerful computational tool that uses deep learning algorithms to predict protein structures with remarkable accuracy. Alphafold multimer could be used to predict protein–protein interaction, which is efficient, cost-effective, and less time-consuming [165]. Despite getting the interaction through the alphafold, the result needs functional validation. Investigating the regulatory processes that control the CYP genes’ expression and function is another important area of research. A comprehensive understanding of the regulatory networks governing the expression of CYP would result from identifying the transcription factors, cis-regulatory elements, microRNAs, and signaling pathways involved. The integrated omics approach has tremendous potential in crop breeding to bridge the gap between mitigating plant stresses and achieving food security through increased production. Integrating data from different omics layers allows for a systems biology approach, enabling researchers to build comprehensive models of crop systems. These models can simulate plant responses to different stresses, predict crop performance under various conditions, and optimize breeding strategies accordingly. In recent years, several CYPs have been investigated as potential candidate genes for biotechnological uses. Despite plant CYP characterization and genetic engineering improvements, there is still a significant gap between current research and the technology’s commercialization. By incorporating the resistant trait in the cultivar, crop productivity could be increased and used for commercial purposes. We can improve our knowledge of CYPs in plants, elucidate their functional roles in various biological processes, and open the door for developing crop varieties with enhanced growth, productivity, and resistance to biotic and abiotic stresses.

## Figures and Tables

**Table 2:** List of several CYP genes in hormone biosynthesis.

Plant Species	CYP Genes	Functions	References
*A. thaliana*	*CYP79B2*, *CYP79B3*, *CYP83*	Biosynthesis of auxin	[33]
	*CYP707A*	ABA regulation	[82]
	*CYP735A1*, *CYP735A2*	Biosynthesis of trans-zeatins	[83]
	*CYP88A*, *CYP714A*	Gibberellin metabolic pathways	[84]
	*CYP707A*	Abscisic acid pathways	[85]
	*AtCYP74A*	Biosynthesis of jasmonic acid	[86]
*O. sativa*	*CYP93B*, *CYP93E*, *CYP93G*	Flavonoid biosynthesis	[65]
	*CYP701A8*, *CYP714B*	Gibberellin metabolic pathways	[87,88]
*Hordeum vulgare*	*CYP707A*	Modulation of abscisic acid	[85]
*S. lycopersicum*	*CYP90B3*, *CYP724B2*	Functions in BR biosynthetic pathway	[89]
*V. vinifera*	*CYP90D1*	BR biosynthesis	[90]

## Data Availability

Not applicable.
